# Cumulative evidence for association between genetic polymorphisms and esophageal cancer susceptibility: A review with evidence from meta‐analysis and genome‐wide association studies

**DOI:** 10.1002/cam4.1972

**Published:** 2019-02-21

**Authors:** Jie Tian, Caiyang Liu, Guanchu Liu, Chunjian Zuo, Huanwen Chen

**Affiliations:** ^1^ Department of Cardiothoracic Surgery The First Affiliated Hospital of Chongqing Medical University Chongqing China

**Keywords:** esophageal cancer, genetic polymorphisms, genome-wide association study, meta‐analyses, susceptibility

## Abstract

An increasing number of publications had reported the association between single‐nucleotide polymorphisms (SNPs) and esophageal cancer (EC) risk in the past decades. Results from these publications were controversial. We used PubMed, Medline, and Web of Science to identify meta‐analysis articles published before 30 July 2018, that summarize a comprehensive investigation for cumulative evidence of genetic polymorphisms of EC and its subtype risk. Two methods, Venice criteria and false‐positive report probability (FPRP) tests, were used to assess cumulative evidence of significant associations. At last, 107 meta‐analyses were considered to be in conformity with the inclusion criteria, yielding 51 variants associated with EC or esophageal squamous cell carcinoma (ESCC). Thirty‐eight variants were considered to be nominally significant associated with risk of EC or ESCC, whereas the rest showed non‐association. In additional, five variants on five genes were rated as strong cumulative epidemiological evidence for a nominally significant association with EC and ESCC risk, including *CYP1A1* rs1048943, *EGF *rs444903, *HOTAIR *rs920778, *MMP2* rs243865, and *PLCE1 *rs2274223, 10 variants were rated as moderate, and 18 variants were rated as weak. Additionally, 17 SNPs were verified noteworthy in six genomewide association studies (GWAS) using FPRP methods. Collectively, this review offered a comprehensively referenced information with cumulative evidence of associations between genetic polymorphisms and EC and ESCC risk.

## 1 INTRODUCTION

Esophageal cancer (EC) is a digestive tract carcinoma and remains the sixth leading cause of a carcinoma‐associated deaths worldwide.^1^, ^2^ In 2015 in the USA, there had been an approximate 16 980 new patients had EC and 15 590 patients died of it,^3^ and its overall five‐year survival rate was only about 20%.^4^, ^5^, ^6^, ^7^ Esophageal squamous cell carcinoma (ESCC) and esophageal adenocarcinoma (EADC) were two different common pathological types of EC, and they were caused by different risk factors. For ESCC, the risk factors were smoking, alcoholic beverages, socioeconomic status (SES), polycyclic aromatic hydrocarbons (PAHs), betel quid, diet quality, low fruit and vegetable intake, micronutrients, pickled vegetables, hot food and beverages and so on. While gastroesophageal reflux disease, Barrett's esophagus and smoking tobacco have been verified as risk factors for EADC. Although these environmental factors were considered to be risk factors for EC, epidemiological and etiological studies have shown that the role of genetic variants was also needed to be considered.^8^

Over the past few decades, a large number of candidate genes association studies^9^, ^10^, ^11^, ^12^ were performed to explore the relationship between gene polymorphisms and EC risk. However, due to the small sample size and inadequate statistical power, the results were instability. Meta‐analysis could present more credible results and stronger statistical power through integrating individual study findings,^13^, ^14^ and more than 100 meta‐analyses had been performed in recent years. Most of the results for same variant, however, were inconsistent. For *EGF *rs4444903, Xu et al^15^ performed a meta‐analysis and found that the variant rs4444903 could decrease the risk of EC (OR = 0.73, 95% CI = 0.61‐0.86), whereas Li et al^16^ found the variant rs4444903 could increase the risk of EC (OR = 1.17, 95% CI = 1.09‐1.25). For *TP53* rs1042522, Zhao et al performed a meta‐analysis and found that the variant rs1042522 could increase the risk of EC (OR = 1.20, 95% CI = 1.06‐1.36), whereas Jiang et al found that the variant rs1042522 had an opposite association with EC risk (OR = 0.73, 95% CI = 0.57‐0.94).^17^ GWAS could screen the sequence variation in the human genome and identify SNPs related to human diseases,^18^ and they extended our understanding of associations between genetic variations and cancer risk.^19^ To date, GWAS that have two stages (discovery and replication) have identified to be a commonly powerful and successful tool in the identification of genetic variants associated with susceptibility to complex human diseases or phenotypes.^20^, ^21^

As early as 2008, Dong et al^22^ reported that three variants were significantly related to EC by assessing the FPRP of meta‐analysis. In recent years, more than 100 meta‐analyses had been performed. When GWAS method is applied, gene mutations or susceptibility loci were identified to have relationship with many diseases.^23^ In 2010, of the 18 SNPs, Wang LD et al^24^ summarized two identified susceptibility loci (10q23 and 20p13) associated with ESCC risk. Meanwhile, Abnet et al^25^ found variants on PLCE1 gene associated with ESCC risk and Wang et al^24^ found that the gene, C20orf54, had significant association with ESCC risk in Chinese population. Later, Jin et al^26^ found consistent associations two loci (6p21.1 and 7p15.3) with EC risk in both GWAS and replication stages. In 2013, Levine et al^27^ added three new susceptibility loci (3p13, 9q22, and 19p13) for EADC. In following years, Chang et al^28^ found another two variants on 13q22.1.

Although more than 100 meta‐analyses and several GWAS with association between genetic variants and EC susceptibility had been performed, these results of different studies for same variant were inconsistent, indicating the possibility of false‐positive associations. Ioannidis et al indicated that mechanisms for summarizing and assessing genetic epidemiological evidence require periodic updates of all appropriate association studies based on widely accepted assessment criteria.^29^ However, the current literature still lacks an updated comprehensive assessment report covering all possible variants of multiple genes with EC risk.

Therefore, we attempt to collect cumulative evidence of associations between genetic variants and EC risk from published meta‐analyses and GWAS, and evaluate these associations, which may offer referenced information for further investigation of genetic risk factors for EC and its subtype.

## 2 METHOD

### 2.1 Literature search strategy and criteria for inclusion

The following items were used in search process: (“esophageal”) and (“cancer” or “adenocarcinoma” or “carcinoma” or “tumor” or “squamous cell carcinoma”) and (“meta‐analysis” or “Meta‐analysis” or “systematic review” or “literature review”) and (“genetic association” or “Genetic” or “SNP” or “polymorphism” or “single nucleotide polymorphism” or “genotype” or “variant” or “variation” or “mutation” or “susceptibility”). Additionally, we also checked all the relevant references to find other potential meta‐analyses that could offer relevant data.

Meta‐analysis articles met to the following criteria: (a) The publications were in English; (b) cancer type was EC or including subtypes; (c) the patients with EC were diagnosed by pathological or histological examination; (d) sample size was not fewer than 1000; (e) they were studied of EC incidence/susceptibility (rather than mortality or survival rate). The following criteria should be met for screening the SNPs in GWAS on PubMed: (a) The publications were in English; (b) cancer type was EC, which includes all the subtype of EC; (c) the patients with EC were diagnosed by pathological or histological examination; (d) the studies included two phases (discovery and replication); (e) OR and 95% CI were provided and the less than cutoff of 1 × 10^‐8 ^of *P* value was considered statistically significant; (f) they were studied of EC incidence/susceptibility (rather than mortality or survival rate).

### 2.2 Data extraction

Data were extracted by J.T and checked by two authors (C.L and GL). Information extracted from each eligible publication in GWAS included PMID of article, first author, publishing year, gene name, genetic variant, ethnicity of participants, the amount of subjects (cases and controls), minor allele frequency (MAF), OR, 95% CI, *P* value. In meta‐analysis, the following data were collected: first author, publishing year, gene name, genetic variant, OR and 95% CI,the number of studies, the number of subjects (cases and controls), ethnicity, I‐square, test for heterogeneity (*Q* test) between studies,^30^ and the test for publication bias (Egger's test).^31^ I‐square refers to the percentage of variation across studies due to heterogeneity. We referenced the Cochran's *Q* test^32^ to evaluate heterogeneity between studies. Generally, the cutoff *P* value used for between‐study test of heterogeneity (*Q*‐test) was 0.10. *P* value >0.10 represents little heterogeneity while *P* value <0.10 indicates the presence of heterogeneity. We attempted to extract information of EC, including ESCC and EADC; however, the results showed that almost all of the articles were studied for EC and ESCC, not for EADC. Therefore, we were unable to conduct assessment of EADC, and then, we just evaluated cumulative epidemiological evidence for EC and ESCC. In addition, the eligible studies reported two major ethnicities, Asian and Caucasian. Majority of the meta‐analyses included a combination of two or more ethnicities, which were defined as “diverse populations.” Therefore, we extracted the information of diverse population, when applicable, Asian and Caucasian population were also extracted. Multiple studies concerning the same SNP reported conflicting results due to varied sample sizes and the selection of different association models. Therefore, given study quality and result credibility, we selected the most recently published study with the greatest number of and most integrated participants and the standardized report of the genetic association study based on guidelines of the Human Genome Epidemiology Network for systematic review of genetic association studies^33^ and Preferred Reporting Items for Systematic Reviews and Meta‐Analyses (PRISMA).^34^ In addition, articles usually offered different genetic models; therefore, the additive model (see Table S1) was considered as the priority model for data extraction and evaluation in order to reduce selection bias. Specifically, the rest models were also used when additive model was not usable. For variant name, the most recent gene names were used to identify the different variants. An association was considered to be statistically significant if the 95% CI excluded 1.0 or if the reported *P* value was <0.05.

### 2.3 Assessment of cumulative evidence

Venice criteria were applied to assess the epidemiological credibility of significant associations identified by meta‐analysis.^29^, ^35^ Credibility was rated as strong, moderate, or weak (grade A, B, or C) according to three elements: amount of evidence, replication of association, and protection from bias. The first element was evaluated by the total of alleles or genotypes among cases and controls which was divided into three groups: >1000, 100‐1000, and fewer than 100 (representing A, B and C, respectively). Although certain test allele or genotype amounts were not provided, we could obtain the MAF from database of SNP on NCBI and further calculate the amounts. Association replication was calculated using heterogeneity statistics assigned one of three grades, as follows: grade A (*I* ^2 ^< 25%), grade B (25% < *I* ^2 ^< 50%), or grade C (*I* ^2 ^> 50%). Protection from bias was mainly determined by sensitivity analysis and a series of bias tests including publication bias, small‐study bias, as well as an excess of significant findings (see Table S2). Briefly, protection from bias was graded as A if there was no observable bias, and bias was unlikely to explain the presence of the association, B if bias could be present, or C if bias was evident or was likely to explain the presence of the association. Assessment of protection from bias also considered the magnitude of association; a score of C was assigned to an association with a summary OR < 1.15, unless the association had been replicated prospectively by several studies with no evidence of publication bias (ie, GWAS or GWAS meta‐analysis from collaborative studies). Nevertheless, the associations did not participant in grading if the information was insufficient for assessment. For cumulative epidemiological evidence, all three elements were A would be considered as strong evidence, a C for any grade were weak, the rest of combinations were moderate.

We performed a false‐positive report probability (FPRP) assay with a prior probability of 0.001 and an FPRP cutoff value of 0.2 to uncover potential false‐positive results among significant associations and evaluate whether these associations should be omitted, and we used the statistical power to detect an odds ratio of 1.5 for alleles with an elevated risk in FPRP calculations, as suggested by Wacholder et al.^36^ Statistical power and FPRP values were calculated by the Excel spreadsheet which was offered on Wacholder's website. If the calculated FPRP value was below the prespecified noteworthiness value of 0.2, we would consider the association noteworthy, indicating the association might be true. Value for FPRP was assigned to three groups: <0.05, 0.05‐0.2, >0.2 (representing strong, moderate and weak, respectively). Cumulative evidence was upgraded from moderate to strong or from weak to moderate base on FPRP <0.05. Conversely, cumulative evidence was downgraded from strong to moderate or from moderate to weak base on FPRP >0.2.

## 3 RESULTS

### 3.1 Description of search results and characteristics of the studies

**Figure 1:**
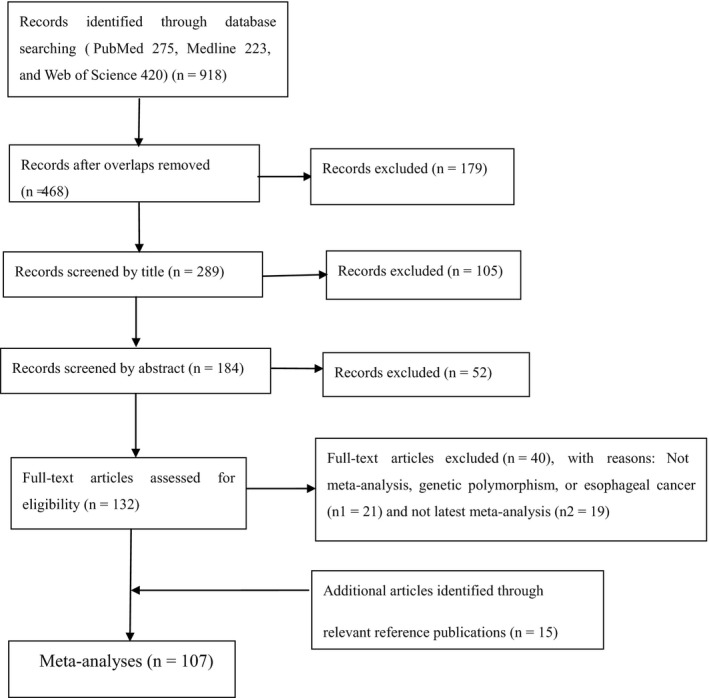
Provides selection of studies

As presented in Figure 1, our search yielded a total of 918 publications. Of these, 179 publications were excluded due to overlaps, 157 irrelevant articles were excluded for reading the title or abstract, 21 articles were excluded due to not meta‐analysis, genetic polymorphism, or esophageal cancer, 19 articles were excluded due to not latest meta‐analysis. In addition, 15 additional articles identified through relevant reference publications. At last, 107 meta‐analyses were eligible for criteria in our review. On the basis of the data that extracted from the method mentioned above, yielding 51 SNPs associated with EC or ESCC susceptibility (45 SNPs in 41 genes, six SNPs in miRNA). We used the PubMed to identify the SNPs in GWAS. As a result, 17 SNPs were identified in six GWAS studies (15 SNPs are in 10 genes and two SNPs are near one gene or between genes).

### 3.2 Significant association in meta‐analyses and GWAS

For 51 SNPs identified by meta‐analysis, 38 SNPs had statistically significant association with EC or ESCC susceptibility.^37^, ^38^, ^39^, ^40^, ^41^, ^42^, ^43^, ^44^, ^45^, ^46^, ^47^, ^48^, ^49^, ^50^, ^51^, ^52^, ^53^, ^54^, ^55^, ^56^, ^57^, ^58^, ^59^, ^60^, ^61^, ^62^, ^63^, ^64^, ^65^, ^66^, ^67^, ^68^, ^69^, ^70^, ^71^, ^72^, ^73^, ^74^ Meta‐analysis results showed that there were 27 SNPs associated with increased EC or ESCC risk, whereas 11 SNPs decreased the risk of EC or ESCC risk. In addition, 17 SNPs were evaluated using the additive model. Ten SNPs were evaluated using the dominant model and another 11 SNPs were assessed using recessive or homozygous model because the additive model was not available.

As presented in Table 1, cumulative epidemiological evidence was graded for 38 significant associations among the main meta‐analyses. Venice criteria were firstly used to assess these associations. Strong, moderate, and weak evidence were assigned to four, seven, and 17 SNPs for EC, and were assigned to one, three, and two SNPs for ESCC, respectively. Next, cumulative evidence were upgraded from moderate to strong for *CYP1A1 *rs1048943, *PLCE1 *rs2274223, *MMP2 *rs243865 in EC and *HOTAIR* rs920778 in ESCC, from weak to moderate for *ADH1B* rs1229984 and *COX‐2* rs20417 in EC, based on FPRP <0.05. Cumulative evidence were downgraded from strong to moderate for *IL‐18 *−607C>A, *MMP1* rs1799750, *SLC52A3* rs13042395 and *MDM2* rs2279744 in EC, and *TNF‐α* rs1800629, *C20orf54 *rs13042395, *microRNA124* rs531564 in ESCC, from moderate to weak for *GSTT1* null/present, *XRCC1* rs1799782, *Hsa‐mir* rs3746444, *hOGG1* rs1052133, *STK15* rs2273535 in EC, and *microRNA‐34b/c* rs4938723, *NAT2* rapid/slow in ESCC, based on FPRP >0.2. Finally, five SNPs on five genes were rated as strong for cumulative epidemiological evidence of association by combining Venice criteria and FPRP results, including *CYP1A1* rs1048943, *EGF* rs444903, *MMP2* rs243865, *PLCE1* rs2274223 for EC and *HOTAIR* rs920778 for ESCC. Seven SNPs with EC and three with EC were rated as moderate. Sixteen with EC and two with ESCC were rated as weak. Great discrepancy between the calculated amount and the true amount makes the grade arduous to determine. Therefore, calculated amounts of less than 3000 were not included for assessment in MAFs obtained from the dbSNP.

**Table 1:** Statistically significant variants from meta‐analyses, false‐positive report probabilities (FPRP), and cumulative epidemiological evidence

PMID	Gene (variant)	Cancer type	Year	Comparison	Ethnicity	OR (95% CI)	Publication bias/heterogeneity	*I* ^2 ^(%)	No. of studies	Cases/control	Number of test allele or genotype (calculated value according to MAF)	Maf	Venice Criteria^a^	Venice Grade	Power Or of 1.5	FPRP values at prior probability of 0.001 at power OR of 1.5	Cumulative epidemiological evidence^b^	Ref.
CYP1A1																		
25048966	CYP1A1 exon7 (rs1048943)	EC	2014	Dominant	Diverse	1.49 (1.33‐1.66)	*P* = 0.815/0.036	38.30%	18	6165 (2552/3613)	2863	0.2281	ABA	Moderate	0.548	0.000	Strong	37
		EC	2014	Dominant	Asian	1.48 (1.33‐1.66)	*P* = 0.925/0.026	44.20%	15	5431 (2381/3050)	2703	0.2598	ABA	Moderate	0.591	0.000	Strong	37
		EC	2014	Dominant	Caucasian	1.50 (0.87‐2.59)	*P* = 0.537/0.254	25.20%	3	734 (171/563)	Na	Na	Na	Na	Na	Na	Na	37
CYP1A1																		
25886559	CYP1A1 (rs4646903)	EC	2015	Additive	Asian	1.25 (1.04‐1.51)	*P* = 0.550/0.000	67.10%	12	3161 (1359/1802)	2645	0.4001	ACA	Weak	0.971	0.955	Weak	38
		ESCC	2015	Additive	Asian	1.17 (1.04‐1.32)	0.544^c^/0.055^c^	47.4%^c^	9	2384 (1027/1357)	1897	0.3868	ABA	Moderate	1.000	0.915	Weak	38
ERCC2 751																		
25748732	ERCC2 (rs13181)	EC	2015	Dominant	Diverse	1.30 (1.07‐1.57)	*P* = No/<0.05	80.00%	21	14 832 (6581/8251)	5570	0.2424	ACA	Weak	0.931	0.873	Weak	39
		EC	2015	Dominant	Asian	1.27 (1.04‐1.56)	*P* = No/<0.05	66.7%^c^	12	7265 (3338/3927)	1778	0.1179	ACA	Weak	0.944	0.960	Weak	39
		ESCC	2015	Dominant	diveres	1.27 (1.04‐1.55)	*P* = 0.229/0.000^c^	66.7%^c^	13	8111 (3351/4760)	2385	0.1673	ACA	Weak	0.949	0.952	Weak	39
		EADC	2015	Dominant	diveres	1.16 (0.87‐1.55)	Na	Na	7	5122 (1726/3396)	Na	Na	Na	Na	Na	Na	Na	39
21667112	ERCC2 (rs1052559)	EC	2012	Homozygous	Asian	2.45 (1.10‐5.44)	*P* = 0.83/0.355	7.7%^c^	4	2352 (1093/1259)	28	0.0436	CAA	Weak	0.114	0.996	Weak	40
		EADC	2012	Homozygous	diveres	1.26 (1.02‐1.56)	*P* = 0.277/0.054	54%^c^	6	4341 (1281/3060)	593	0.3565	BCA	Weak	0.945	0.973	Weak	40
		ESCC	2012	Homozygous	diveres	1.32 (0.85‐2.06)	Na	Na	6	3185 (1294/1281)	Na	Na	Na	Na	Na	Na	Na	40
ERCC2																		
25209371	ERCC2 (rs238406)	ESCC	2014	Dominant	Asian	1.24 (1.04‐1.49)	*P* = No/0.017	Na	1	2257 (1126/1131)	1534	0.424	AXA	Na	0.979	0.957	Na	41
Fas																		
24598538	Fas (rs2234767)	EC	2014	Recessive	Diverse	1.58 (1.16‐2.13)	*P*=>0.05/0.089	58.70%	3	2660 (1126/1534)	Na (0‐490)	0.1841^d^	XCA	Na	0.367	0.880	Na	42
GSTP1																		
25280543	GSTP1 (rs1695)	EC	2015	Additive	Caucasian	1.146 (1.031‐1.275)	*P* = 0.901/0.175	30.40%	9	3289 (1198/2019)	2333	0.3479	ABC^e^	Weak	1.000	0.925	Weak	43
		ESCC	2015	Additive	Caucasian	1.041 (0.956‐1.134)	Na	Na	15	Na	Na	Na	Na	Na	Na	Na	Na	43
		EADC	2015	Additive	Caucasian	1.096 (0.971‐1.237)	Na	Na	10	Na	Na	Na	Na	Na	Na	Na	Na	43
HOTAIR																		
27791260	HOTAIR (rs920778)	ESCC	2017	Recessive	Asian	2.525 (1.921‐3.320)	*P*=>0.396/0.368	0.1%^c^	3	4221 (2071/2150)	259	0.2105	BAA	Moderate	0.000	0.000	Strong	44
IL‐18																		
26214646	IL‐18 (−607C>A)	EC	2015	Dominant	Diverse	1.29 (1.00‐1.66)	*P* = 0.088/0.70	0.00%^c^	2	1749 (1305/444)	1175	0.4561	AAA	Strong	0.879	0.982	Moderate	45
MMP1																		
23644699	MMP1 (rs1799750)	EC	2013	Dominant	Diverse	1.47 (1.18‐1.82)	*P* = 0.127/0.78	0.00%	3	1936 (856/1080)	1457	0.5181	AAA	Strong	0.574	0.415	Moderate	46
MnSOD																		
23679296	MnSOD (rs4880)	EC	2013	Dominant	Na	1.74 (1.36‐2.22)	*P* = 0.61/0.64	0.00%	4	1529 (620/909)	Na (628‐1256)	0.4107^d^	XAA	Na	0.116	0.067	Na	47
MTHFR C677T																		
24606463	MTHFR (rs1801133)	EC	2014	Additive	Asian	1.19 (1.06‐1.34)	*P* = 0.667/<0.001	Na	14	6633 (2808/3825)	6491	0.4567	AXA	Na	1.000	0.803	Na	48
NAT2																		
24595082	NAT2 (rapid/slow)	ESCC	2013	slow vs rapid	Asian	1.35 (1.03‐1.77)	*P* = 0.805/0.093	49.70%	5	1534 (441/1093)	402	0.118	BBA	Moderate	0.777	0.975	Weak	49
hOGG1																		
23909557	hOGG1 (rs1052133)	EC	2013	Recessive	diveres	1.40 (1.12‐1.74)	*P* = 0.140/0.176	27.40%	12	5984 (2363/3621)	586	0.2813	BBA	Moderate	0.733	0.767	Weak	50
		EC	2013	Recessive	Asian	1.51 (1.15‐1.96)	*P* = 0.140/0.22	29.00%	6	2461 (1123/1338)	408	0.3961	BBA	Moderate	0.48	0.803	Weak	50
		ESCC	2013	Recessive	diveres	1.86 (1.36‐2.53)	*P* = 0.140/0.73	0.00%	3	1271 (589/682)	200	0.3512	BAA	Moderate	0.085	0.474	Weak	50
		EADC	2013	Recessive	Caucasian	1.08 (0.69‐1.67)	Na	Na	3	2611 (1189/1422)	Na	Na	Na	Na	Na	Na	Na	50
TNF‐α																		
27821804	TNF‐α (rs1800629)	ESCC	2016	Dominant	Diverse	1.19 (1.00‐1.41)	*P* = No/0.405	3.30%	8	4469 (1144/3325)	1297	0.1582	AAA	Strong	0.996	0.978	Moderate	51
PLCE1																		
25422186	PLCE1 (rs2274223)	EC	2014	Dominant	Diverse	1.30 (1.16‐1.46)	*P* = No/0.0003	68.00%	12	22 935 (9912/13 023)	Na (6846‐13 692)	0.2985^d^	ABA	Moderate	0.992	0.009	Strong	52
		EC	2014	Dominant	Asian	1.39 (1.24‐1.57)	*P* = No/0.009	61.00%	10	19 263 (8737/10 526)	Na (5750‐11 500)	0.2985^d^	ABA	Moderate	0.89	0.000	Strong	52
STK15 T>A																		
25452806	STK15 (rs2273535)	EC	2015	Recessive	Asian	1.19 (1.03‐1.38)	*P* = 0.835/0.24	26.00%	6	3725 (1767/1958)	1260	0.5523	ABA	Moderate	0.999	0.955	Weak	53
C20orf54																		
26154995	C20orf54 (rs13042395)	ESCC	2015	Additive	Diverse	0.95 (0.90‐0.99)	*P* = 0.604/0.33	13.00%	7	88 324 (29 922/58 402)	Na (19 855)	0.1124^d^	AAA	Strong	1.000	0.937	Moderate	54
CASP8 −652 6N																		
28915630	CASP8 −652 6 N (rs3834129)	EC	2017	Additive	Asian	0.81 (0.72‐0.92)	*P* = 0.002/0.712	0.00%^c^	3	2608 (1412/1196)	1338	0.3020	AAC	Weak	0.999	0.542	Weak	55
CYP2E1																		
23226753	CYP2E1 (RSqI/PstI)	EC	2012	Additive	Asian	0.64 (0.50‐0.81)	*P* = Na/<0.01	80.00%	17	4226 (1663/4266)	2279	0.3016	ACX	Na	0.367	0.358	Na	56
Hsa‐mir																		
25433484	Hsa‐mir‐499 (rs3746444)	EC	2014	Additive	Asian	0.80 (0.66‐0.98)	*P *= >0.05/0.75	0.00%^c^	2	1358 (669/689)	498	0.2017	BAA	Moderate	0.961	0.97	Weak	57
MicroRNA 34																		
28415817	MicroRNA (rs4938723)	ESCC	2017	Homozygous	Asian	0.787 (0.638‐0.972)	*P* = 0.622/0.345	9.50%	4	4650 (2226/2424)	433	0.3245	BAA	Moderate	0.938	0.965	Weak	58
MicroRNA 124																		
26171202	MicroRNA‐124 (rs531564)	ESCC	2015	Additive	Asian	0.87 (0.77‐0.98)	*P* = No/0.69	0.00%	3	4077 (1964/2113)	1257	0.1621	AAA	Strong	1.000	0.956	Moderate	59
MMP2																		
23644699	MMP2 (rs243865)	EC	2013	Dominant	Asian	0.67 (0.55‐0.80)	*P* = 0.072/0.59	0.00%	3	2781 (1050/1731)	668	0.1416	BAA	Moderate	0.522	0.018	Strong	60
SLC52A3																		
27600099	SLC52A3 (rs13042395)	EC	2016	Recessive	Diverse	0.84 (0.76‐0.93)	*P* = 0.357/0.738	0.00%	11	26 956 (10 027/16 929)	2009	0.3592	AAA	Strong	1.000	0.44	Moderate	61
ADH1B																		
27450204	ADH1B (rs1229984)	EC	2016	Additive	Diverse	0.67 (0.59‐0.76)	*P* = No/<0.00001	87.00%	20	23 148 (9158/13 990)	30 676	0.6983	ACA	Weak	0.531	0.000	Moderate	62
		EC	2016	Additive	Asian	0.66 (0.57‐0.75)	*P* = No/<0.00001	88.00%	18	22 038 (8687/13 351)	30 595	0.7299	ACA	Weak	0.439	0.000	Moderate	62
ALDH2																		
25848305	ALDH2 (rs671)	EC	2015	Homozygous	Diverse	0.69 (0.48‐0.98)	*P* = 0.682/<0.001	74.80%	31	24 707 (8510/16 197)	1439	0.2446	ACA	Weak	0.576	0.985	Weak	63
		EC	2015	Homozygous	Asian	0.68 (0.60‐0.79)^c^	*P* = 0.682/0.000^c^	74.3%^c^	30	23 481 (8130/15 351)	1429	0.2573	ACA	Weak	0.602	0.001	Moderate	63
CCND1																		
24944806	CCND1 (rs603965)	EC	2014	Recessive	Diverse	1.33 (1.03‐1.73)	*P* < 0.001/0.000	72.10%	11	5343 (2111/3232)	1471	0.5203	ACC	Weak	0.815	0.976	Weak	64
		EC	2014	Recessive	Asian	1.22 (0.93‐1.60)	*P* < 0.001/0.0228	30.70%	4	Na	Na	Na	Na	Na	Na	Na	Na	64
		EC	2014	Recessive	Caucasian	1.44 (0.97‐2.14)	*P* < 0.001/0.000	80.70%	7	Na	Na	Na	Na	Na	Na	Na	Na	64
		ESCC	2014	Recessive	diveres	1.28 (0.93‐1.75)	Na	Na	Na	Na	Na	Na	Na	Na	Na	Na	Na	64
		EADC	2014	Recessive	Caucasian	1.59 (0.69‐3.70)	Na	Na	Na	Na	Na	Na	Na	Na	Na	Na	Na	64
COX‐2																		
21304218	COX‐2 (rs20417)	EC	2011	Additive	Diverse	1.45 (1.23‐1.71)	*P* = 0.922/0.003	76.00%	4	3779 (1562/2217 )	638	0.0753	BCA	Weak	0.656	0.015	Moderate	65
		EC	2011	Additive	Asian	1.71 (1.37‐2.17)	*P* = 0.922/Na	Na	2	2686 (1200/1486 )	307	0.0451	BXA	Na	0.141	0.067	Na	65
EGF																		
23403233	EGF (rs4444903)	EC	2013	Additive	Diverse	1.38 (1.20‐1.59)	*P* = 0.476/0.997	0.00%	3	1713 (779/934)	1659	0.4534	AAA	Strong	0.876	0.009	Strong	66
ERCC2 Asp312Asn																		
25356096	ERCC2 (rs1799793)	EC	2014	Dominant	Diverse	1.14 (1.03‐1.27)	*P* = 0.096/1.00	0.00%	15	9940 (3928/6012)	2981	0.1865	AAC^e^	Weak	1.000	0.946	Weak	67
		EC	2014	Dominant	diveres	1.12 (0.99‐1.27)	Na	Na	Na	Na	Na	Na	Na	Na	Na	Na	Na	67
		EC	2014	Dominant	diveres	1.20 (0.99‐1.47)	Na	Na	Na	Na	Na	Na	Na	Na	Na	Na	Na	67
GSTM1																		
26855551	GSTM1 (null/present)	EC	2016	Additive	Diverse	1.33 (1.12‐1.57)	*P* = 0.0873/<0.000001	77.00%	37	11 949 (4572/7377)	5478	0.2197	ACA	Weak	0.922	0.449	Weak	68
		EC	2016	Additive	Asian	1.53 (1.26‐1.86)	*P* = 0.0873/0.000001	77.2%^c^	27	8406 (3336/5070)	3814	0.2099	ACA	Weak	0.421	0.045	Moderate	68
GSTT1																		
23244092	GSTT1 (null/present)	EC	2012	Additive	Asian	1.26 (1.05‐1.52)	*P* = 0.270/0.04	42.70%	15	3842 (1626/2216)	1686	0.2128	ABA	Moderate	0.966	0.942	Weak	69
MDM2																		
24844868	MDM2 (rs2279744)	EC	2015	Additive	Diverse	0.88 (0.81‐0.96)	*P* = 0.83/0.263	22.80%	6	4915 (1899/3016)	4762	0.4741	AAA	Strong	1.000	0.799	Moderate	70
		EC	2015	Homozygous	Asian	0.7 (0.58‐0.84)	*P* = 0.94/0.539	0.00%	5	4150 (1059/2562)	1076	0.4881	AAA	Strong	0.7	0.152	Strong	70
MTHFR A1298C																		
23679298	MTHFR (rs1801131)	EC	2013	Recessive	Diverse	1.843 (1.414‐2.402)	*P* = 0.801/0.435	0.00%	6	3693 (1302/2391)	246	0.2388	BAA	Moderate	0.064	0.087	Moderate	71
		EC	2013	Recessive	Asian	3.997 (1.614‐9.900)	*P* = 0.801/0.409	0.00%	4	1652 (598/1054)	20	0.1456	CAA	Weak	0.017	0.994	Weak	71
		EC	2013	Recessive	Caucasian	1.693 (1.280‐2.240)	*P* = 0.801/0.936	0.00%	2	2037 (704/1333)	226	0.3132	BAA	Moderate	0.198	0.534	Weak	71
		ESCC	2013	Recessive	diveres	2.57 (1.76‐3.76)	Na	Na	Na	Na	Na	Na	Na	Na	Na	Na	Na	71
		EADC	2013	Recessive	diveres	1.47 (1.07‐2.01)	Na	Na	Na	Na	Na	Na	Na	Na	Na	Na	Na	71
NQO1 607C>T																		
28203294	NQO1 (rs1800566)	EC	2017	Additive	Diverse	1.13 (1.01‐1.26)	*P *= >0.05/0.000	74.40%	13	5385 (2357/3028)	Na (3110)	0.2889^d^	ACC^e^	Weak	1.000	0.965	Weak	72
TP53																		
23844939	TP53 (rs1042522)	EC	2013	Additive	Diverse	1.146 (1.106‐1.293)	*P* = 0.481/<0.001	70.10%	14	11 492 (4184/7308)	11 550	0.4698	ACC^e^	Weak	1.000	0.964	Weak	73
		EC	2013	Additive	Asian	1.194 (1.031‐1.384)	*P* = 0.499/<0.001	70.40%	11	7614 (2317/5297)	6714	0.4188	ACA	Weak	0.999	0.949	Weak	73
XRCC1 Arg194Trp																		
23543084	XRCC1 (rs1799782)	EC	2013	Recessive	Asian	1.332 (1.093‐1.624)	*P* = 0.902/0.074^c^	42.30%	10	5290 (1946/3344)	457	0.2929	BBA	Moderate	0.88	0.839	Weak	74
		ESCC	2013	Recessive	Asian	1.43 (1.16‐1.75)^c^	*P* = 0.872/0.315^c^	14.3%^c^	9	5068 (1840/3228)	429	0.2893	BAA	Moderate	0.679	0.432	Weak	74

Na, Not available; No significant publication bias/heterogeneity was not found, diversee: two or more ethnicities were reported in the meta‐analysis.

a Venice Criteria grades are evidence of amount, replication of the association, and protection from bias.

b Cumulative epidemiological evidence as graded by combination of results from Venice Creteria and FPRP.

c The information is calculated according to the data provided in the article since the article did not present (such as *I* ^2^,OR, publication bias and heterogeneity).

d The MAF is obtained from dbSNP database.

e The grade of C is given because the OR value is less than 1.15 and the association is not replicated by GWAS or GWAS meta‐analysis.

The associations including 17 SNPs identified in six GWAS studies were presented in Table 2.^24^, ^26^, ^27^, ^28^, ^75^, ^76^, ^77^ Eleven SNPs listed in chart had associations with increased EC risk. Opposite association was found in six SNPs,^26^, ^27^, ^28^, ^75^ all of which were regarded as noteworthy based on FPRP method. The way of Venice Criteria was not applicable to GWAS which has not enough datasets even regarding the two‐step GWAS including discovery and replication phases as individual studies,^29^ we did not further evaluate these results. In addition, two variant (rs13042395 on *C20orf54* and rs2274223 on *PLCE1*) were performed both in meta‐analysis and in GWAS.

**Table 2:** Statistically significant variants from GWAS

PMID	Gene	Year	Variant	ethnicity	OR (95% CI)	MAF^b^	*P*‐value^c^	Cases/control	Power OR of 1.5	FPRP values at prior probability of 0.001 at power OR of 1.5	Ref.
20729852	PLCE1	2010	rs3765524	Asian	1.35 (1.22‐1.49)	0.258/0.207	1.74E‐09	5417 (2115/3302)	0.982	0.000	75
	PLCE1	2010	rs2274223	Asian	1.43 (1.37‐1.49)	Na	7.46E‐56	5417 (2115/3302)	0.842	0.000	75
20729853	C20orf54	2010	rs13042395	Asian	0.86 (0.82‐0.90)	Na	1.21E‐11	22 336 (9053/13 283)	1.000	0.000	24
	HEATR	2012	rs4785204	Asian	1.24 (1.18‐1.29)	Na/0.26	2.24E‐20	20 787 (10 123/10 664)	1.000	0.000	24
22960999	HEATR	2012	rs7206735	Asian	1.20 (1.15‐1.26)	Na/0.28	1.97E‐16	20 787 (10 123/10 664)	1.000	0.000	76
	HAP1	2012	rs6503659	Asian	1.27 (1.20‐1.34)	Na/0.13	2.73E‐10	20 787 (10 123/10 664)	1.000	0.000	76
	XBP1	2012	rs2239815	Asian	1.18 (1.13‐1.23)	Na/0.37	3.88E‐15	20 787 (10 123/10 664)	1.000	0.000	76
	ST6GAL1	2012	rs2239612	Asian	1.21 (1.15‐1.27)	Na/0.19	5.74E‐15	20 787 (10 123/10 664)	1.000	0.000	76
	SMG6	2012	rs17761864	Asian	1.21 (1.14‐1.28)	Na/0.14	2.21E‐11	20 787 (10 123/10 664)	1.000	0.000	76
	PTPN2	2012	rs2847281	Asian	1.20 (1.14‐1.26)	Na/0.16	2.49E‐11	20 787 (10 123/10 664)	1.000	0.000	76
	CHEK2	2012	rs4800983	Asian	1.27 (1.21‐1.34)	Na/0.20	1.94E‐22	20 787 (10 123/10 664)	1.000	0.000	76
	CHEK2	2012	rs1033667	Asian	1.25 (1.19‐1.30)	Na/0.25	4.85E‐22	20 787 (10 123/10 664)	1.000	0.000	76
24121790	KLF5	2015	rs1924966	Asian	0.84 (0.80‐0.89)	0.35/0.40	1.37E‐10	12 356 (6177/6179)	1.000	0.000	27
26315552	KLF5	2015	rs115797771	Asian	0.69 (0.62‐0.78)	0.05/0.06	2.32E‐10	12 356 (6177/6179)	0.709	0.000	28
	KLF5	2015	rs58090485	Asian	0.69 (0.62‐0.77)	0.05/0.07	1.23E‐10	12 356 (6177/6179)	0.731	0.000	28
	TMEM173^a^	2014	rs7447927	Asian	0.85 (0.82‐0.88)	Na	7.72E‐20	30 286 (15 667/14 619)	1.000	0.000	28
25129146	ATP1B2^a^	2014	rs1642764	Asian	0.88 (0.85‐0.91)	Na	3.10E‐13	30 281 (15 474/14 807)	1.000	0.000	77

Na, Not available.

a The SNP is near the genes in brackets.

b Minor Allele Frequency (MAF) in Case/Control.

c The *P* values are all <1.00E‐08.

### 3.3 Nonsignificant association in meta‐analyses

We performed statistical power analyses to determine the stability of the associations. In our meta‐analysis results, 13 variants were not significantly associated with EC or ESCC risk.^37^, ^42^, ^60^, ^78^, ^79^ The variants (Arg399Gln on *XRCC1) *with sample sizes >10 000 were also not significantly associated with EC; further investigations for this variant may not be fruitful. Certain variants presented with relatively small sample sizes; as such, the evidence for nonsignificant (see Table S3) was considered unstable.

### 3.4 Inconsistency among meta‐analyses

Twenty variants presented inconsistent results in our review (see Table S4). All in all, 13 SNPs were deemed to have significant association, as follows: *ADH1B *rs1229984, *ALDH2* rs25848305, *MDM2* rs2279744, *XRCC1* rs1799782, *TP53* rs1042522, *CCND1* rs603965, *COX‐2 *rs20417, *EGF *rs4444903, *ERCC2 *rs1799793, *GSTM1* null/present, *GSTT1* null/present, *MTHFR* rs1801131 and *NQO1* rs1800566. Seven SNPs were deemed to have nonassociation: *EPHX1* (rs2234922 and rs1051740), *Fas* 1800682, *XPA *rs1800975, *microRNA146 *rs2910164, *microRNA196* rs11614913, *XRCC1* rs25487. The results of two variants, *EPHX1 *(Try113His and His139Arg)^81^, ^83^, ^84^ and *XRCC1* (Arg399Gln and Arg194Trp),^74^, ^86^, ^87^ should be prudently interpreted due to similar sample size.

## 4 DISCUSSION

This review collates a comprehensive investigation for cumulative evidence of genetic polymorphisms of EC and its subtype risk. We extracted relevant useful information from meta‐analyses and GWAS to support a comprehensive assessment for further evaluation. Using FPRP tests and Venice criteria, we scored strong, moderate, or weak cumulative evidence as credibility and strength of an association with cancer susceptibility. Five SNPs on five genes with strong evidence of association were identified, including *CYP1A1* rs1048943, *EGF* rs444903, *MMP2* rs243865, *PLCE1* rs2274223 for assessing risk of EC and *HOTAIR* rs920778 for ESCC. Ten variants were found to have moderate evidence of association with EC or ESCC risk, and 18 variants weak evidence.

CYP1A1, located on chromosome 15‐q22, is an isozyme of cytochrome P450 and encods aryl hydrocarbon hydroxylase (AHH) which may combine with DNA to form adducts via a series of biochemical reactions. The ultimate carcinogens converted from the DNA adducts were considered to be associated with the development of EC.^37^, ^90^ SNP (rs1048943) was rated as strong evidence of association with a 1.49‐fold increased risk of EC in overall population based on over 6000 sample size. This SNP triggers an increase in the enzymatic activity and increases the activation of enzyme induction, thus may accelerate cancer development.^37^, ^91^ In our subgroup analysis, this SNP increased EC risk based on 5431 sample size in Asians, whereas nonassociation was found in Caucasians based on 734 sample size. More studies of this variant in Caucasians or other ethnic groups should be performed.

EGF, located on chromosome 4q25‐q27,^92^, ^93^ participates in the process of proliferation and differentiation of cells^94^ and promotes gene transcription when EGF binds to its receptor.^95^ Quiet a few studies have identified the G allele promoted the EGF protein expression when EGF binds to its receptor which could interfering DNA folding and further increased susceptibility of a range of human cancers.^96^ Our review showed that the G allele of EGF +61A>G (rs4444903) polymorphism was rated as strong evidence of association with 1.38‐fold increased risk of EC based on 1713 sample size.

MMP‐2 is a sort of zinc‐dependent endopeptidases, which can regulate various cell behaviors such as tumor initiation and growth by modulating cell proliferation, apoptosis and angiogenesis.^97^, ^98^ The SNP (rs243865), located in the promoter region of the MMP‐2, disrupts an Sp1‐type promoter site (CCACC box) and then affects MMP‐2 expression or activity, which was considered to be associated with development of cancer condition.^99^ Our review showed that the SNP (rs243865) was rates as strong evidence of association with EC risk (OR = 0.67, 95% CI = 0.55‐0.80) under dominant model. All studies were performed on a single ethnic group (Asian), and we recommend expanding studies on this variant to other ethnic groups.

PLCE1, located on chromosome 10q23, participate in cell growth, differentiation, gene expression and oncogenesis.^100^, ^101^ Our review showed that this SNP (rs2274223) was rated as strong evidence of association with increased risk of EC for a common A to G transition of PLCE1 that may increase expression of PLCE1 protein^102^, ^103^ in a diverse population based on a sample size of over 20 000. In our subgroup analysis, this SNP was found significant association with EC risk in Asians, whereas nonassociation was found in Caucasians. Although the mechanism for ethnic differences is still unclear, one possible reason is due to differences in genetic backgrounds and in the environmental and lifestyle context.

HOTAIR, located on chromosome 12q13.13, might transform normal cells to malignant.^44^ As Zhang et al^105^ suggested, the TT genotype was nominally significant related to ESCC susceptibility among Chinese population when compared with the rs920778 CC genotype.^44^ In our review, the SNP (rs920778) was rated as strong evidence of true association with ESCC risk for the T allele of HOTAIR that may induce genome‐wide retargeting of polycomb‐repressive complex 2, trimethylates histone H3 lysine‐27 (H3K27me3) and deregulation of multiple downstream genes which participated in development and progression of ESCC^105^ based on 4221 sample size (OR = 2.525, 95% CI = 1.921‐3.320). Again, all studies were performed on a single ethnic group (Asian), and we again recommend expanding studies on this polymorphism to other ethnic groups.

There are six variants showing moderate evidence of association in our review, all of which downgraded from strong to moderate: *IL‐8 *−607C>A, *MMP‐1* rs1799750, *TNF‐α* rs1800629, *MicroRNA124* rs531564, *SLC52A3* rs13042395 and *MDM2* rs2279744 based on a high FPRP (> 0.2). The FPRP method considers the *P* value, prior probability, and statistical power of the test; as we calculated FPRP at prior probability of 0.001 and used the statistical power to detect an odds ratio of 1.5 for alleles with an elevated risk in FPRP calculations, certain otherwise significant associations may have been excluded. Previous studies using different prior probabilities have classified their results as more noteworthy. Further investigations on these six variants may be necessary to analyze their associations in greater depth. Additionally, SNP (rs13042395) on *C20orf54* also showing moderate evidence of association with ESCC susceptibility, which downgraded from strong to moderate. This SNP was both verified in GWAS and assessed in meta‐analysis. The association in GWAS, however, may be more statistically significant and convincing when related meta‐analysis results are inconsistent or the association evidences are not strong by combination Venice criteria and FPRP method. The Venice criteria could assess multiple reasons of potential bias such as genotype error or misclassification and ethnicity stratification, which were difficult to perform in meta‐analysis. Therefore, results might be more convincing if different weights in the Venice Criteria were reset.

Cumulative evidence of two variants (*ADH1B* rs1229984 and *COX‐2* rs20417) with risk of EC was upgraded from weak to moderate based on FPRP <0.05. Additional assessment of two variants were necessary, particularly the variant (*COX‐2* rs20417) since sample size of study for this variant are relatively small (a total of 3779 sample size).^65^, ^106^

Thirteen variants were found not to be significantly associated with EC risk, to include nine variants on seven genes and two mRNAs in a sample of approximately 4000 patients, at approximately 85% power to detect an OR of 1.15 under different model for a variant with MAF of 20%. The MAFs of the aforementioned seven variants were almost all over 0.3 despite sample sizes >4000. We can safely conclude, therefore, that these seven variants are unlikely to be associated with EC (see Table S5). Further investigations evaluating these seven variants will probably not yield meaningful results with regards to EC.

Certain limitations do apply to this report. Although we did a comprehensive literature search, some articles may have been missed. A variability in sample size presented among different studies; the smaller sizes may have impacted the credibility of the data. The evaluated data extracted from only one source which may be main cause of bias. Finally, only the susceptibility/incidence between genetic variants and EC risk were evaluated; however, other roles of genetic polymorphisms such as tumor progression, metastasis, drug resistance for EC were not be assessed due to lack of data or information. Despite the limitations of our method, we believe that our study, as an updated summary and evaluation of the existing literature reporting genetic predisposition of EC, is of value for further genetic studies.

This review evaluated the cumulative epidemiological evidence of significant associations by combining the Venice criteria and FPRP results which identified five SNPs having strong evidence of true association. Collectively, our review provides referenced information for further investigation into genetic susceptibility of EC and ESCC.

## CONFLICT OF INTEREST

None declared.
