# Supplementary material for: Cumulative evidence for association between genetic polymorphisms and esophageal cancer susceptibility: A review with evidence from meta‐analysis and genome‐wide association studies
Source: Cancer Med. 2019 Feb 21;8(3):1289–305. doi: 10.1002/cam4.1972 (PMC6434199; doi:10.1002/cam4.1972)
Supplement: Supplementary file 1 [file CAM4-8-1289-s001.doc]

**Supporting Information Table 1:** **Supplementary notes for genetic model**

The complete data structure of genetic polymorphism study

| Genotype amount | | | |
| --- | --- | --- | --- |
| Genotype type | A A | A B | BB |
| Case group | a_n_ | b_n_ | c_n_ |
| Control group | d_n_ | e_n_ | f_n_ |

AA:Wild homozygous AB: Heterozygous mutant BB: Mutant homozygous

n: indicating the Nth study.

For a SNP, two alleles, A and B, could be presented. Specifically, A was considered as wild type,meanwhile, B was considered mutant type. Therefore, there may be three genotypes, AA, AB, BB, respectively, in population. Suppose there were three genotypes of the subjects, we could assign a,b,c to AA, AB, BB in case group ,and d,e,f to AA, AB, BB in control group, respectively. The table above could offer additional explanation.

In meta-analysis for SNPs, polygenic model was used to decrease probabilities of type I error. The following genetic models may be used in our study: 1) Additive model (i.e. B vs A); 2) Dominant model (BB+BA vs AA); 3) Recessive model (BB vs BA+AA); 4) Homozygous model ( BB vs AA). Specifically, the additive model was used first, and the rest models were also used when additive model was not usable.
