# Supplementary material for: Cumulative evidence for association between genetic polymorphisms and esophageal cancer susceptibility: A review with evidence from meta‐analysis and genome‐wide association studies
Source: Cancer Med. 2019 Feb 21;8(3):1289–305. doi: 10.1002/cam4.1972 (PMC6434199; doi:10.1002/cam4.1972)
Supplement: Supplementary file 2 [file CAM4-8-1289-s002.doc]

**Supporting Information:** **Supplementary notes for details of protection from bias and supplementary table for genetic variants significantly associated with EC risk in meta-analyses .**

**Protection from bias**

A: No observable bias and bias was unlikely to explain the presence of the association.

B: No obvious bias may affect the presence of the association, but there is considerable missing information on the identification of evidence.

C: Bias is demonstrable or is likely to explain the presence of the association.

The Venice criteria include an extensive checklist for sources of bias in different settings. The checklist has different considerations depending on whether the evidence comes from retrospective meta-analyses of published data or prospective GWAS and replication studies from collaborative consortia with harmonization of data collection and analysis.

General checks for bias that have been adopted for meta-analysis are: (1) Association lost with exclusion of first study; (2) Association lost with exclusion of studies deviated from HWE; (3) Small effect size of association (i.e., OR < 1.15); (4) Evidence of publication bias (p < 0.05 in Egger's test); (5) Evidence of small-study effect; (6) Evidence is presented for an excess of individual studies with significant finding.

**Supporting Information Table 2: Details of protection from bias for genetic variants significantly associated with EC risk in meta-analyses**

Note: HWE= Hardy-Weinberg equilibrium. No: significant publication bias was not found. NA: Not available.

| **Gene**  **(Variants)^Ref^** | **Cancer risk**  OR (95% CI) | **Venice criteria grade** | **Protection from bias** | **Reason for bias** | **Reason for bias exemption** | ***p* value for publication bias** | **OR<1.15** | ***p* value for small study bias** | **Association lost with exclusion of first study** | **Deviation from HWE** |
| --- | --- | --- | --- | --- | --- | --- | --- | --- | --- | --- |
| CYP1A1(rs1048943)^37^ | 1.49 (1.33-1.66) | ABA | A | NA | NA | 0.815 | No | NA | NA | NA |
| CYP1A1 (rs4646903)^38^ | 1.25 (1.04-1.51) | ACA | A | NA | NA | 0.550 | No | NA | NA | NA |
| ERCC2 (rs13181)^39^ | 1.30 (1.07-1.57) | ACA | A | NA | NA | No | No | NA | NA | NA |
| ERCC2 (rs1052559)^40^ | 2.45 (1.10-5.44) | CAA | A | NA | NA | 0.83 | No | NA | NA | NA |
| ERCC2 (rs238406)^41^ | 1.24 (1.04-1.49) | AXA | A | NA | NA | No | No | NA | NA | NA |
| Fas (rs2234767)^42^ | 1.58(1.16-2.13) | XCA | A | NA | NA | > 0.05 | No | NA | NA | NA |
| GSTP1(rs1695)^43^ | 1.146(1.031-1.275) | ABC | **C** | **Low OR** | NA | 0.901 | Yes | NA | NA | NA |
| HOTAIR(rs920778)^44^ | 2.525(1.921-3.320) | BAA | A | NA | NA | >0.396 | No | NA | NA | NA |
| IL-18(-607C>A)^45^ | 1.29(1.00-1.66) | AAA | A | NA | NA | 0.088 | No | NA | NA | NA |
| MMP1(rs1799750)^46^ | 1.47(1.18-1.82) | AAA | A | NA | NA | 0.127 | No | NA | NA | NA |
| MnSOD(rs4880)^47^ | 1.74(1.36-2.22) | XAA | A | NA | NA | 0.61 | No | NA | NA | NA |
| MTHFR(rs1801133)^48^ | 1.19(1.06-1.34) | AXA | A | NA | NA | 0.667 | No | NA | NA | NA |
| NAT2 (rapid/slow)^49^ | 1.35(1.03-1.77) | BBA | A | NA | NA | 0.805 | No | NA | NA | NA |
| hOGG1(rs1052133)^50^ | 1.40(1.12-1.74) | BBA | A | NA | NA | 0.140 | No | NA | NA | NA |
| TNF-α(rs1800629)^51^ | 1.19(1.00-1.41) | AAA | A | NA | NA | No | No | NA | NA | NA |
| PLCE1(rs2274223)^52^ | 1.30(1.16-1.46) | ABA | A | NA | NA | No | No | NA | NA | NA |
| STK15(rs2273535)^53^ | 1.19(1.03-1.38) | ABA | A | NA | NA | 0.835 | No | NA | NA | NA |
| C20orf54(rs13042395)^54^ | 0.95(0.90-0.99) | AAA | A | NA | NA | 0.604 | NA | NA | NA | NA |
| CASP8 -652 6N(rs3834129)^55^ | 0.81(0.72-0.92) | AAC | **C** | **publication bias** | NA | 0.002 | NA | NA | NA | NA |
| CYP2E1(RSqI/PstI)^56^ | 0.64(0.50-0.81) | ACX | X | NA | NA | NA | NA | NA | NA | NA |
| Hsa-mir-499(rs3746444)^57^ | 0.80(0.66-0.98) | BAA | A | NA | NA | > 0.05 | NA | NA | NA | NA |
| MicroRNA (rs4938723)^58^ | 0.787(0.638-0.972) | BAA | A | NA | NA | 0.622 | NA | NA | NA | NA |
| MicroRNA-124(rs531564)^59^ | 0.87(0.77-0.98) | AAA | A | NA | NA | No | NA | NA | NA | NA |
| MMP2 (rs243865)^60^ | 0.67(0.55-0.80) | BAA | A | NA | NA | 0.072 | NA | NA | NA | NA |
| SLC52A3(rs13042395)^61^ | 0.84(0.76-0.93) | AAA | A | NA | NA | 0.357 | NA | NA | NA | NA |
| ADH1B(rs1229984)^62^ | 0.67(0.59-0.76) | ACA | A | NA | NA | No | NA | NA | NA | NA |
| ALDH2(rs671)^63^ | 0.69(0.48-0.98) | ACA | A | NA | NA | 0.682 | NA | NA | NA | NA |
| CCND1(rs603965)^64^ | 1.33(1.03-1.73) | ACC | **C** | **publication bias** | NA | <0.001 | No | NA | NA | NA |
| COX-2(rs20417)^65^ | 1.45(1.23-1.71) | BCA | A | NA | NA | 0.922 | No | NA | NA | NA |
| EGF(rs4444903)^66^ | 1.38(1.20-1.59) | AAA | A | NA | NA | 0.476 | No | NA | NA | NA |
| ERCC2(rs1799793)^67^ | 1.14(1.03-1.27) | AAC | **C** | **Low OR** | NA | 0.092 | Yes | NA | NA | NA |
| GSTM1(null/present)^68^ | 1.33(1.12-1.57) | ACA | A | NA | NA | 0.0873 | No | NA | NA | NA |
| GSTT1(null/present)^69^ | 1.26(1.05-1.52) | ABA | A | NA | NA | 0.270 | No | NA | NA | NA |
| MDM2(rs2279744)^70^ | 0.88(0.81-0.96) | AAA | A | NA | NA | 0.83 | NA | NA | NA | NA |
| MTHFR(rs1801131)^71^ | 1.843(1.414-2.402) | BAA | A | NA | NA | 0.801 | No | NA | NA | NA |
| NQO1(rs1800566)^72^ | 1.13(1.01-1.26) | ACC | **C** | **Low OR** | NA | > 0.05 | Yes | NA | NA | NA |
| TP53(rs1042522)^73^ | 1.146(1.106-1.293) | ACC | **C** | **Low OR** | NA | 0.481 | Yes | NA | NA | NA |
| XRCC1(rs1799782)^74^ | 1.332(1.093-1.624) | BBA | A | NA | NA | 0.902 | No | NA | NA | NA |
